# The Case of Mrs. Hannah Lowe, Aged about 32 Years, Who Was Nearly Five Months Gone with Child, and Died of a Mortification in the Bladder, &c.

**Published:** 1800-05

**Authors:** Thomas Dray

**Affiliations:** Hythe, Kent


### *To the Editors of the Medical and Physical Journal.*

GENTLEMEN,

IF you can see, in the following case, that any useful inferences may be drawn therefrom, as a future guide to practitioners, and think it worthy an insertion in your very estimable publication, I shall think myself happy in thus adding my mite to the stock of so valuable a branch of knowledge as the practice of physic. I am,

GENTLEMEN,

Your most humble servant,

THOMAS DRAY.

*Hythe, Kent,*
*April* 8, 1800.

---

### *The Case of Mrs. Hannah Lowe, aged about* 32 *Years, who was nearly five Months gone with Child, and died of a Mortification in the Bladder, &c.*

LATE at night of the 10th of last February, Mrs. Hannah Lowe was taken with pains so like to those of labour, that she expected an abortion would take place immediately. I was called upon to attend her early the next morning, when she informed me, that she supposed herself to be about four months gone with child, and that her pains had been regular and bearing. Finding no discharge, ab utero, and that she had passed but but little urine, an enema was ordered, and, ſoon afterwards, an anodyne. Some hours afterwards, as the fæcal diſcharge had been trifling from the enema, a gentle laxative was given, by which her inteſtines were well emptied. It was plainly to be perceived, on an examination per vaginam, that there was a great bearing down, which led me to ſuppoſe, with the woman herſelf, that an abortion would not be long before it took place. She had external hæmorrhoids to ſo great a ſize, that I judged it neceſſary to puncture them. In a day or two after, on being informed that ſhe now made ſo little water that it amounted to nearly a ſuppreſſion of it, I paſſed the catheter, and drew off more than three pints of high-coloured urine, which greatly relieved her for a time; and, the next day, I drew off nearly the ſame quantity, but much paler. Obſerving that her ſeeming labour-pains recurred in proportion as the bladder became diſtended, I conſidered it proper to draw off her water, morning and evening, daily. In this manner ſhe went on, without any material alteration, always complaining of a pain and tenderneſs of the abdomen, till about the tenth day, when her urine became ſo very offenſive that I could hardly bear its ſtench. The uterus was forced down much lower into the pelvis, and the os internum, with the cervix uteri, clearly to be perceived in a ſtraight direction towards the os externum. It was very plain to me now, from the fœtor of the urine, and the turbidneſs of it, (ſeveral times before) that the bladder was in a very diſeaſed ſtate; and I began to doubt whether there was any defect in the uterus. To be brief, the woman kept on in this way (except that the abdomen got more and more enlarged, in an even and regular manner, up to the ſcrobiculus cordis, and the pain was much greater) till about the 2d of March, when ſhe was ſeized with a hiccough that increaſed upon her till ſhe died, which was on the 4th of March, in the evening, being the twenty-third day of the diſeaſe. The os tincæ, for three or four days before ſhe expired, was quite at the verge of the os externum, but no diſcharge whatever was perceived to come from the uterus. There was no heat of the ſkin through the whole of this poor woman's caſe, no great drought, or acceleration of her pulſe, till a day or two before the cloſe of it. The alvine diſcharge was, in general, regular, excepting a ſmall time before ſhe died, when ſhe had a frequent diſcharge by ſtool, and likewiſe a vomiting of porraceous bile. The urine diſcharged by the catheter* was very high coloured, and much leſs

* It is to be remarked that the patient never made a drop of water after the firſt two or three days of her illneſs; and, for many days previous to her diſſolution, the point of the catheter was tarniſhed every time of uſing it.

lefs in quantity, for the laft two or three days, and always followed with a bloody, foul mucus Her tongue was clean, the whole time of her difeafe. She got up and dreffed herfelf every day. Her fpirits were, commonly, good; and her fenfes perfect to the laft.

On opening the body, the omentum prefented itfelf in a morbid ftate, adhering to the peritonæum, very generally, through its whole extent, and likewife to the inteftines; and alfo the bladder adhered to the uterus. In fhort, the abdominal vifcera appeared like one mafs, the adhefions being fo general. The bladder was much thickened and enlarged, and contained nearly two quarts of urine, in which was a great quantity of foul mucus, and the whole of it was in a complete ftate of gangrene. The uterus feemed not to be in the leaft difeafed, and, from appearance, the fœtus * could not have been long dead. The whole of the gravid uterus was forced very low down into the pelvis, which (as I have before mentioned) might be plainly perceived, whilft the woman was alive, by examining per vaginam. The kidnies and liver were in a found ftate. A great quantity of fluid was effufed into the cavity of the abdomen.

The principal remarks to be made in this cafe, before and after the death of the patient, feem to me to be thefe: *First*, the feeming labour-pains, and bearing down of the uterus; — *Secondly*, the partial difcharge at firft, and, afterwards, total fuppreffion of urine; — *Thirdly*, the gradual enlargement of the abdomen; — *Fourthly*, the abfence of fever, nearly, through the whole of the difeafe; — *Fifthly*, the great thicknefs of the bladder, and its extremely gangrenous ftate.

As this poor woman had been in a very healthy ftate previous to this attack, (excepting a complaint that fhe had for fome time made to her friends, of an obtufe pain at the region of the bladder, running through to the back, which fhe did not think of confequence enough to confult me upon) I was much at a lofs to account for the caufe of the difeafe; which difeafe, with the principal fymptoms in it, joined to what appeared on diffection, feems to me, now, to have clearly begun in the bladder. This has been, to my mind, fatisfactorily explained fince, by the friends of the patient. They informed me, that frequently in the courfe of the day, fhe was in the habit of reaching over a cheft, and taking a variety of things, fuch as coals, &c. from behind it, placed there for convenience, as the family † had only

---

* The fœtus, by its fize, feemed to agree with the woman's ftatement of the time of her pregnancy.

† The family confifted only of herfelf, her hufband, and one child.

only one room (a chamber) to live in. The preſſure on the bladder,* ſo very often, by the edge of the cheſt, when in a diſtended ſtate, muſt injure it, I make no doubt, to a great degree; and by the preſſure of that viſcus and the inteſtines, at ſuch a time, on the uterus, this latter was forced down lower into the pelvis than natural at this period of its gravidity. The uterus being ſo low down in the pelvis, at the latter end of the diſeaſe, may be explained by the great ſize and diſtenſion of the bladder, as it was not poſſible to draw off the urine entirely, for a day or two previous to her diſſolution. The pains, which alternated like thoſe in labour, were clearly from the diſeaſed ſtate, and diſtenſion of the bladder; and the total ſuppreſſion of urine muſt ariſe chiefly, if not entirely, from the morbid ſtate of it. The gradual enlargement, and conſtant pain in the abdomen, proceeded from the ſlow but ſteady progreſs towards gangrene, which had extended itſelf along the peritonæum, &c. nearly as high up as the ſcrobiculus cordis. But the wonder now comes, why there was not the leaſt ſymptom of fever (excepting near the cloſe of the diſeaſe) when there was ſuch a morbid ſtate of the bladder, &c. and ſuch great adheſions? I muſt confeſs that I am at a loſs to account for a thing ſo extraordinary, but this was the fact,—and it was equally a fact, that the woman's ſpirits and ſtrength kept up in a moſt aſtoniſhing manner, notwithſtanding ſhe ſlept but little during the whole time of her illneſs.

The indications, in this caſe, were not ſuch as to point out the uſe of bleeding, bliſtering, and other antiphlogiſtic means, the only ones which now ſuggeſt themſelves as having the leaſt chance of relieving the patient. The whole of the treatment that was indicated, ſeemed to me to conſiſt in the evacuation of the urine, watching and keeping up the alvine diſcharge, and alleviating the ſymptoms occaſionally.

